# Measuring Gait Stability in People with Multiple Sclerosis Using Different Sensor Locations and Time Scales

**DOI:** 10.3390/s21124001

**Published:** 2021-06-10

**Authors:** Roy Müller, Lucas Schreff, Lisa-Eyleen Koch, Patrick Oschmann, Daniel Hamacher

**Affiliations:** 1 Department of Orthopedic Surgery, Klinikum Bayreuth GmbH, 95445 Bayreuth, Germany; lucas.schreff@klinikum-bayreuth.de (L.S.); lisa-eyleen.koch@klinikum-bayreuth.de (L.-E.K.); 2 Department of Neurology, Klinikum Bayreuth GmbH, 95445 Bayreuth, Germany; patrick.oschmann@klinikum-bayreuth.de; 3 Department of Sports Science, Friedrich Schiller University Jena, 07749 Jena, Germany; daniel.hamacher@uni-jena.de

**Keywords:** MS, 6-min walk, 25-foot walk, local dynamic stability, Lyapunov exponent, fatigue, EDSS

## Abstract

The evaluation of local divergence exponent (LDE) has been proposed as a common gait stability measure in people with multiple sclerosis (PwMS). However, differences in methods of determining LDE may lead to different results. Therefore, the purpose of the current study was to determine the effect of different sensor locations and LDE measures on the sensitivity to discriminate PwMS. To accomplish this, 86 PwMS and 30 healthy participants were instructed to complete a six-minute walk wearing inertial sensors attached to the foot, trunk and lumbar spine. Due to possible fatigue effects, the LDE short (~50% of stride) and very short (~5% of stride) were calculated for the remaining first, middle and last 30 strides. The effect of group (PwMS vs. healthy participants) and time (begin, mid, end) and the effect of Expanded Disability Status Scale (EDSS) and time were assessed with linear random intercepts models. We found that perturbations seem to be better compensated in healthy participants on a longer time scale based on trunk movements and on a shorter time scale (almost instantaneously) according to the foot kinematics. Therefore, we suggest to consider both sensor location and time scale of LDE when calculating local gait stability in PwMS.

## 1. Introduction

Gait analysis is frequently utilized in patients with multiple sclerosis (PwMS). To estimate gait stability or the risk of falling in PwMS, different measures have been used. Besides the calculation of gait variability (e.g., [1,2,3]) or margin of stability (e.g., [4]), the evaluation of local dynamic stability (operationalized through the local divergence exponent, LDE) has been proposed as a common gait stability measure (e.g., [5,6,7,8]).

In particular, local dynamic stability is an approach to evaluate the ability of the locomotor system to recover from small perturbations that occur during walking [9]. An inability of the locomotor system to compensate for such perturbations results in higher LDE and thus, lower walking stability. LDE was therefore proposed as a measure to rate fall risk in PwMS [8,10]. For that matter, the LDE depicts a reasonable construct, predictive, and convergent validity [11]. Previous studies analyzing the LDE in PwMS have shown that e.g., the LDE is greater (lower stability) in PwMS than in healthy participants [2,6]. However, differences in methods of determining LDE, such as different sensor locations (e.g., sacrum, shoulder or foot) or different types of data (e.g., acceleration or gyro), may lead to different results [11,12].

Lizama et al. [6] calculated the LDE for acceleration data of three markers placed on sacrum, shoulder and cervical while walking barefoot on a treadmill at 1.2 m/s. They found that PwMS with no evident gait impairments are less stable than healthy participants. Additionally, they observed a significant main effect of the sensor location, yet no significant interaction. However, sensors placed on the foot as used in studies investigating gait stability of walking in an older population [13] were not taken into account. To be able to compare studies, the effects of different sensor locations on the sensitivity to discriminate PwMS with a varying level of disability (based on their Expanded Disability Status Scale, EDSS, [14]) from healthy subjects should be assessed. This would also be a prerequisite to choose a good sensor location and, therefore, a prerequisite to use LDE in a clinical setting.

Another factor that affects gait and gait stability in PwMS is motor fatigue. To demonstrate motor fatigue in PwMS, a wide range of tests is available. A recently used test is the 6-min walk that measures the total distance a patient can walk in six minutes [15,16,17]. Goldman et al. [16] examined walking speed profiles in PwMS during the walking test and found that they decelerate continuously across the 6-min observation period (calculated per minute). Furthermore, Leone et al. [17] found that more than a third of PwMS experience walking-related motor fatigue during a 6-min walk, with the prevalence being highest in more disabled patients. To investigate the effect of walking-related motor fatigue on changes in local dynamic stability, Arpan et al. [18] examined minute-by-minute changes in LDE (trunk acceleration data) during a 6-min walk in healthy participants and PwMS. They found that (compared to healthy participants) PwMS became less stable over time whereas differences persisted at min-6. However, to the best of our knowledge, the effect of sensor-based walking-related motor fatigue on changes in LDE in PwMS with a varying level of disability has not been investigated so far.

The purpose of the current study was to determine the effect of different sensor locations (trunk, lumbar spine and foot) on the sensitivity to discriminate PwMS with a varying level of disability based on local gait stability (LDE). Furthermore, we want to investigate the effect of walking-related motor fatigue on changes in LDE during the 6-min walk.

## 2. Materials and Methods

### 2.1. Participants

For this study, 86 PwMS and 30 healthy participants were recruited sequentially in the outpatient clinic of the Department of Neurology, Klinikum Bayreuth GmbH, Germany (Table 1; [19]). PwMS were conducted by a scientific assistant during their waiting time in the anteroom of the outpatient clinic and were eligible to participate in case of (i) a verified MS diagnosis [20], (ii) an age between 18–65 years, and (iii) the ability to walk without a walking aid for at least six minutes. They were not included in case of (i) a recent relapse, (ii) severe musculoskeletal or cardiovascular disorders interfering with balance and gait, and (iii) other neurological diseases except MS. Healthy participants were free from any neurological, cardiovascular and major musculoskeletal disease. All participants provided written informed consent. The study was approved by the ethical review board of the Friedrich Schiller University Jena, Germany (2018-1221-BO) and was in accordance with the Declaration of Helsinki.

### 2.2. Procedure

**Figure 1:**
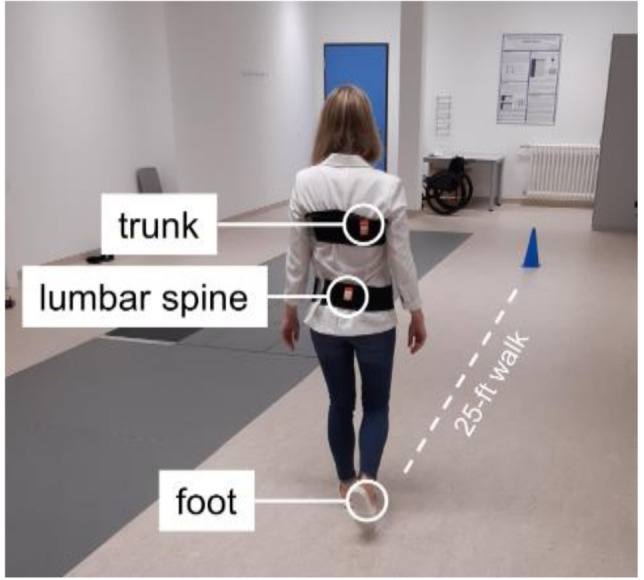
Experimental setup. Both PwMS and healthy participants had to complete a walking test that required them to cover a distance of 25 feet (7.62 m) repeatedly throughout a maximal assessment period of six minutes. Wearable inertial sensors were attached to the forefoot of participants’ dominant foot (foot), and with an elastic belt to the right scapula (trunk) and lumbar spine (at L5 to approximate body center of mass).

Assessments took place in the Klinikum Bayreuth GmbH. Both PwMS and healthy participants had to complete a walking test that required them to cover a distance of 25 feet (7.62 m) repeatedly throughout a maximal assessment period of six minutes as enduring and fast as possible (6-min 25-ft walk; Figure 1). A cone was placed three feet away from each endpoint of the 25-foot distance and participants circled the cones to make their turn back toward the 25-foot distance [21].

To measure local dynamic gait stability, wearable inertial sensors were utilized (MTw2, Xsens Technologies B.V.; sampling rate: 100 Hz, range of measurement of angular velocity: ±1200 deg/s) throughout the walking test. The sensors were attached to the forefoot of participants’ dominant foot (i.e., the foot they would take to kick a ball), and with an elastic belt to the upper trunk (right scapula) and lumbar spine (at L5 to approximate body center of mass; Figure 1). In several studies, the upper trunk sensor was attached to the sternum e.g., [2] or the thoracic spine at T7 or shoulder level e.g., [6,8]. Because we attached the upper trunk sensor with an elastic belt and our experience has shown that in some cases the elastic belt was not close to the body (e.g., in the area of the sternum or the lumbar spine at T7 level), we decided to attach the upper trunk sensor on the right scapula to guarantee that the sensor is tight and does not swing on the belt. However, when considering the upper body as a solid body, the sensor position should not affect the results of LDE measure.

### 2.3. Data Processing

As a measure of local dynamic stability, we analyzed the LDE (local divergence exponent, sometimes referred to as the largest Lyapunov exponent) for each sensor (foot, trunk, and lumbar spine), separately. At first, we determined gait events (e.g., heel-strike) with a reliable algorithm [22,23]. Thereafter the first and the last bout of the 6-min-walking trial were omitted. Furthermore, the first and the last 2.5 m of each bout (acceleration and deceleration phases before and after turning) were excluded from the following calculations.

The LDE was calculated for the remaining first 30, middle 30 and last 30 strides. While for the LDE analysis in healthy adults the use of 150 strides has been supposed to maximize statistical precision [24], the data need also to be quasi-stationary. Since fatiguing effects have been reported for a 6-min-walking test in PwMS (e.g., [15,16]), shorter time series must be analyzed. Furthermore, short time series have also successfully been used to analyze LDE, e.g., 10 strides in children [25].

As a basis to analyze the LDE, we reconstructed a state-space based on the three-dimensional gyroscope (angular velocity) data which resulted in the highest effects in comparing the LDE of older vs. younger adults [13] and was, therefore, used in several studies (e.g., [26,27]). Since we used the calibrated gyroscope data outputted from the proprietary sensor fusion algorithm, we did not additionally implement any protocol to correct for time-dependent sensor errors. The angular velocity data of the 30 strides were interpolated to 3000 time-normalized samples. Thereafter a state-space ‘S(t)’ was reconstructed using the time-delayed embedding method with a fixed time delay of τ = 10 and a fixed embedded dimension of dE = 9 (2 copies of the three-dimensional angular velocity time-series; [11]): S(t) = [ωx(t), ωy(t), ωz(t), ωx(t + τ), ωy(t+τ), ωz(t + τ), ωx(t + 2τ), ωy(t + 2τ), ωz(t + 2τ)], with ‘ω’ representing the angular velocity data and the subscripts the corresponding sensor axis. The time delay τ is in our case fixed to 10.

Afterward, the LDE was calculated with Rosenstein’s and co-workers’ algorithm [28]. Thereto, the divergence curves $d_{j}\left(i\right)$ of each initial nearest neighbors j in the state-space was tracked in time (i) and the average of the logarithm divergence curves (ln $d_{j}\left(i\right)$) was calculated: $\mathrm{div}\left(i\right)=\frac{1}{\Delta t}mean\left(\ln\;d_{j}\left(i\right)\right)$ [28]. In our study, we dropped the scaling factor ($\frac{1}{\Delta t}$) which does not affect the statistical effects. As a measure of LDE short, the slope of the linear least-squares fit to the divergence curve $\mathrm{div}\left(i\right)$ from i = 1 to i = 50 time-normalized samples (approximately 0.5 strides) was determined. As an additional measure of LDE very short, the slope of the linear fit from i = 1 to i = 5 time-normalized samples (τ/2, approximately 5% of a stride) was also calculated. While this last version is not frequently used yet (e.g., in [29,30]), effect sizes might be larger [31]. A large LDE value indicates (a large divergence or) low local dynamic gait stability and vice versa.

### 2.4. Statistical Analysis

Statistical analyses were performed with r (version 4.0.4). Since we wanted to compare different sensor locations and effects caused by the different scales of the LDE-values, we z-transformed the LDE values. Thereafter, the effects of group (PwMS vs. healthy participants) and time (begin, mid, end (reference)) of the 6-min 25-ft walk were analyzed for each LDE measure separately using linear random intercepts models. Since quadratic time effects have been previously reported (e.g., [15]), we also included a quadratic time component as a fixed effect into the models. Additionally, all possible interaction effects were included. Furthermore, the effect of EDSS and time on each LDE measure (excluding the control group) were assessed with linear random intercepts models. Again, a quadratic time component and all possible interaction effects were modeled.

## 3. Results

### 3.1. PwMS vs. Healthy Comparison Group

For LDE short a significant main effect of group was found for the trunk sensor (*p* = 0.047; Table 2). Thus, PwMS walked less stable compared to healthy participants (5–8% higher LDE short values for the trunk sensor in PwMS, Table 2). In contrast to LDE short, for LDE very short a significant main effect of group was found for the foot sensor (*p* < 0.001; Table 2). Thus, the foot kinematics were less stable in PwMS compared to healthy participants (6–8% higher LDE very short values for the foot sensor in PwMS, Table 2). In addition to the group effect, a significant linear main effect of time (‘time’) was only found for LDE short of the lumbar spine sensor (*p* = 0.039; Table 2) for both groups (based on the missing interaction effects). There was no significant group × time or group × time^2^ interaction, neither for LDE short nor for LDE very short (Table 2).

### 3.2. PwMS with a Varying Level of Disability

**Figure 2:**
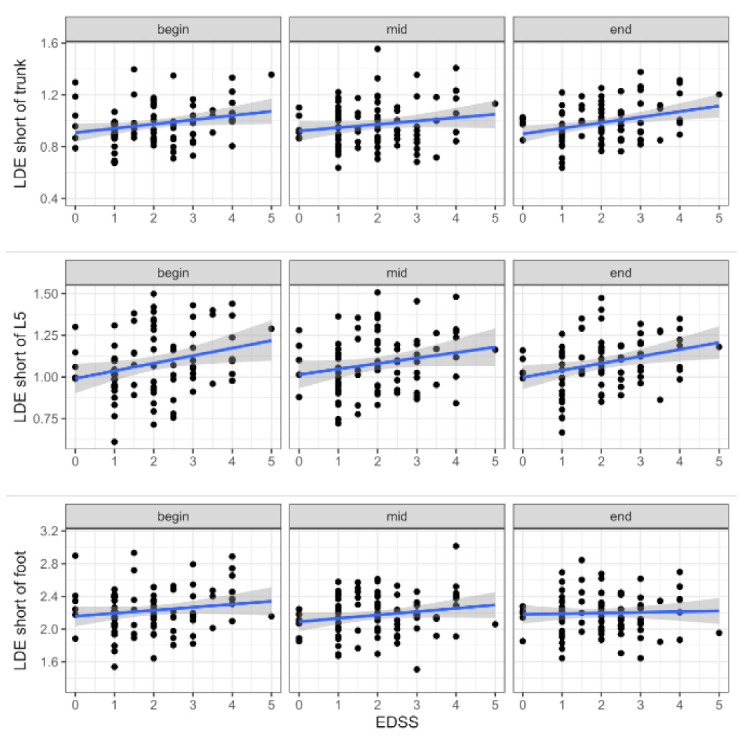
Changes of LDE short over different levels of EDSS, calculated for the remaining first 30 (begin), middle 30 (mid) and last 30 strides (end) during a 6-min 25-ft walk and separated for the foot, lumbar spine (L5), and trunk sensor.

**Figure 3:**
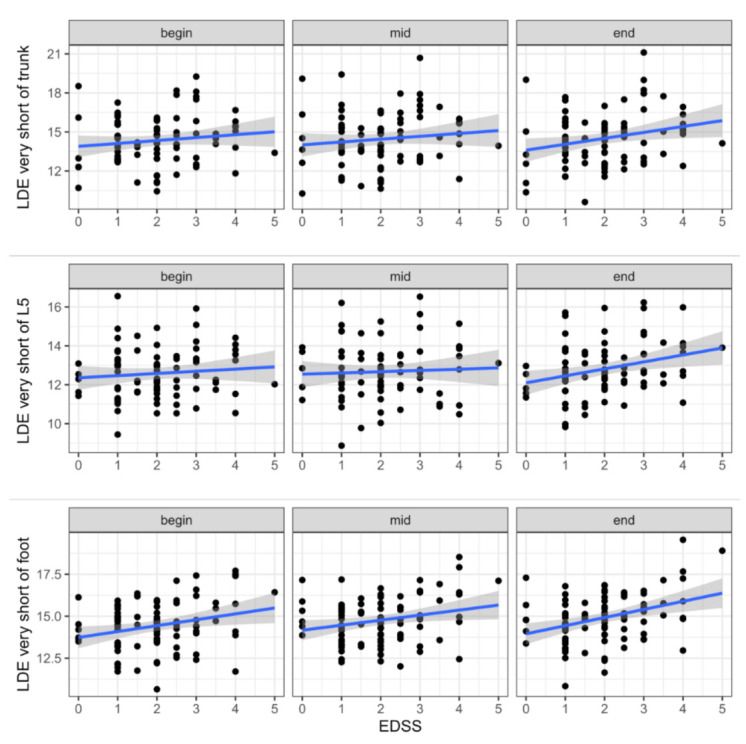
Changes of LDE very short over different levels of EDSS, calculated for the remaining first 30 (begin), middle 30 (mid) and last 30 strides (end) during a 6-min 25-ft walk and separated for the foot, lumbar spine (L5), and trunk sensor.

For LDE short a significant main effect of EDSS was found for the trunk (*p* = 0.012) and lumbar spine sensor (*p* = 0.014; Table 3, Figure 2). More precisely, PwMS with higher EDSS values were less stable. In contrast to LDE short, for LDE very short a significant main effect of EDSS was found for the foot sensor (*p* = 0.004; Table 3, Figure 3), indicating a more stable foot trajectory in healthy participants. In addition to the effect of EDSS, a significant main effect of time^2^ was found for LDE very short of the lumbar spine sensor (*p* = 0.049). The effect of EDSS on LDE very short of the lumbar spine sensor was highest at the last 30 strides, during the 30 strides in the middle the effect diminished. Significant EDSS × time interactions were found for LDE very short of the trunk (*p* = 0.018 (EDSS × time)) and lumbar spine sensor (*p* = 0.002 (EDSS × time), *p* = 0.014 (EDSS × time^2^)). Again, the effect of EDSS on LDE increased to the end of the gait trial.

## 4. Discussion

The primary aim of the current study was to determine the effect of different sensor locations (lumbar spine, trunk and foot) and measures (short, very short) of local gait stability (LDE) on the sensitivity to discriminate PwMS with a varying level of disability. Furthermore, we wanted to investigate the effect of walking-related motor fatigue on changes in LDE during the 6-min walk.

We found that the sensitivity to differentiate PwMS from healthy participants varies with sensor location and LDE measure. More precisely, for LDE short a significant main effect of group (PwMS vs. healthy participants) was found for the trunk sensor, whereas for LDE very short a significant main effect of group was found for the foot sensor (Table 2). The two LDE measures reflect the effect of small perturbations on different time scales: approximately 50% (short) vs. 5% (very short) of the stride time (mean stride time can be found between 0.96 s for healthy participants and 1.01 s for PwMS; see Table 1). Perturbations seem to be better compensated in the healthy comparison group on a longer time scale based on trunk movements and on a shorter time scale (almost instantaneously) according to the foot kinematics. Probably, some mechanisms to compensate perturbations occur nearly instantly or in a short period within the lower extremities, for example, when preparing the leg for ground contact by an adequate adjustment of muscle (pre-) activation [32,33] or joint stiffness [34]. On the other hand, some trunk-based mechanisms might need more time to affect stability, e.g., recovery movements from perturbations due to arm-swing [35] or directing the ground reaction forces to an intersection point above the center of mass [36,37]. While this seems to be intuitive, it is also very speculative and needs to be proven in further studies.

In addition to the Lyapunov exponent, also the effect of other techniques for analyzing local dynamic stability could be investigated in future. For example, the Hurst exponent is considered as a global measure when long-term memory is represented in signals [38]. Moreover, a correlation between Hurst and Lyapunov exponent has already been proven [39]. However, in line with our results, different effects on LDE short and LDE very short (0–3%) have recently been reported [30]. Here the LDE (0–50%) based on the foot kinematics discriminated between healthy younger and healthy older adults but the LDE (0–3%) differed between foot conditions (barefoot vs. shoes). The authors supposed that biomechanical features of the shoe compensate for very small perturbation at least in part instantaneously during the stance phase. Since the LDE very short reflects the ability to compensate small perturbations on a different time-scale, it might be an interesting measure of gait stability that also might reflect different mechanisms to compensate perturbations.

Since gait parameters (e.g., walking speed and stride length [40]) and local dynamic stability change with age (e.g., [41]), some of the differences between PwMS and healthy participants can be explained by age-related effects. Thus, the significant age difference of seven years in our study (Table 1) is probably a confounding factor in the comparison between PwMS and healthy participants. However, a closer look at the varying level of disability (based on their Expanded Disability Status Scale, EDSS) shows that within PwMS, several LDE values also increased with disease progression. Here, significant main effects of EDSS can be observed again in the foot sensor for LDE very short (Table 3, Figure 3) and in the trunk (and lumbar spine) sensor for LDE short (Table 3, Figure 2). This is in accordance with our results on PwMS vs. healthy controls.

Our results indicate that PwMS walked less stable than healthy participants. This is consistent with previous studies analyzing the LDE in PwMS (e.g., [2,6]). However, in these studies, the LDE was calculated over the entire duration of the measurement. This does not seem to play an important role for PwMS at early stages of MS or for PwMS without symptoms of fatigue. Nevertheless, since more than a third of PwMS experience walking-related motor fatigue during a 6-min walk (with the prevalence being highest in more disabled patients; [17]), the effects of motor fatigue should also be included in the LDE measurement. To investigate the effect of walking-related motor fatigue on changes in LDE, Arpan et al. [18] investigated minute-by-minute changes in LDE of trunk acceleration data (estimated from the slope of the mean log divergence curves from 0 to 0.5 strides, comparable to LDE short of the present study) during a 6-min walk in healthy participants and PwMS. They found that PwMS became less stable over time whereas differences persisted at minute-6. However, they did not investigate the effect of sensor-based walking-related motor fatigue on changes in LDE in PwMS with a varying level of disability. In accordance with Arpan et al. [18], we found a significant main effect of fatigue (time) for LDE short of the lumbar spine sensor when comparing PwMS and healthy participants, but no significant (group × time) interaction (Table 1). One of the reasons for this could be that, compared to Arpan et al. [18], PwMS of our study have a lower EDSS and thus, lower effects of fatigue. Additionally, within PwMS we found a significant main effect of fatigue (time^2^) for LDE very short of the lumbar spine sensor and significant (EDSS × time) interactions for LDE very short of the trunk and lumbar spine sensor (Table 2). More precisely, in fatigued PwMS, LDE changes become more obvious (on a shorter time scale) in the trunk and lumbar spine sensor.

Some limitations should be mentioned. First, all participants were wearing their own shoes and different biomechanical shoe features might affect the gait pattern, as discussed. This confounder is not controlled in this study. However, patients are commonly examined using their own shoes, and walking with unfamiliar shoes might also affect the gait pattern. Therefore, in our study, we chose to analyze the participants while wearing their own shoes. Second, the mean age for PwMS group was seven years higher than the healthy participants group (Table 1). Thus, the age difference in our study is possibly another confounding factor in the comparison of PwMS and healthy participants.

## 5. Conclusions

The purpose of the current study was to determine the effect of different sensor locations and time scales on the sensitivity to discriminate PwMS with a varying level of disability. We found that both sensor location and time scale have an influence on LDE measure and thus, the sensitivity to differentiate PwMS from healthy participants. Moreover, perturbations seem to be better compensated in healthy participants and less disabled PwMS (lower EDSS) on a longer time scale based on trunk movements and on a shorter time scale (almost instantaneously) according to the foot kinematics. Therefore, we suggest to consider both sensor location and time scale of LDE when calculating local gait stability in PwMS as an important prerequisite for the use of LDE in a clinical setting.

## Figures and Tables

**Table 1:** Participants’ demographics, Expanded Disability Status Scale (EDSS, range: 0–5) and spatiotemporal measures of gait (walking speed, stride length, stride time).

	Healthy	PwMS	*p*-Value
	mean ± sd	mean ± sd	
Age [years]	34.7 ± 8.9	41.7 ± 11.4	0.003
Height [cm]	172.8 ± 8.5	170.9 ± 8.1	0.368
Weight [kg]	71.7 ± 11.9	79.2 ± 18.3	0.085
Sex [f/m]	21/9	62/24	0.827
EDSS		2.00 ± 1.10	
Walking speed [m/s]			
begin	1.68 ± 0.20	1.46 ± 0.23	0.000
mid	1.66 ± 0.19	1.42 ± 0.23	0.000
end	1.67 ± 0.17	1.42 ± 0.23	0.000
Stride length [m]			
begin	1.60 ± 0.16	1.45 ± 0.17	0.000
mid	1.59 ± 0.16	1.44 ± 0.17	0.000
end	1.60 ± 0.15	1.44 ± 0.17	0.000
Stride time [s]			
begin	0.96 ± 0.06	1.00 ± 0.08	0.005
mid	0.96 ± 0.06	1.02 ± 0.08	0.001
end	0.96 ± 0.06	1.02 ± 0.08	0.000
*N*	30	86	

**Table 2:** LDE measures for healthy participants and PwMS. Mean ± standard deviation of measured local divergence exponent (LDE) for healthy participants and PwMS, separated for the first (begin), middle (mid) and last (end) 30 steps. β: model parameter estimate after z-transformation of the LDE values.

		Group		Model Estimates					
		Healthy	PwMS	Intercept	Group	Time	Time^2^	Group × Time	Group × Time^2^
Trunk	begin	0.92 ± 0.13	0.97 ± 0.16	b = −0.27 β = 0.92	b = 0.37	b = 0.11	b = −0.17	b = 0.41	b = 0.55
(short)	mid	0.92 ± 0.15	0.97 ± 0.17		β = 0.06	β = 0.02	β = −0.03	β = 0.07	β = 0.09
	end	0.92 ± 0.17	0.99 ± 0.15		*p* = 0.047	*p* = 0.928	*p* = 0.891	*p* = 0.771	*p* = 0.696
Lumbar spine	begin	1.01 ± 0.14	1.08 ± 0.19	b = −0.22 β = 1.03	b = 0.30	b = 2.08	b = −0.06	b = −2.12	b = 0.14
(short)	mid	1.03 ± 0.16	1.08 ± 0.18		β = 0.05	β = 0.36	β = −0.01	β = −0.37	β = 0.02
	end	1.05 ± 0.17	1.08 ± 0.16		*p* = 0.124	*p* = 0.039	*p* = 0.948	*p* = 0.074	*p* = 0.907
Foot	begin	2.21 ± 0.26	2.23 ± 0.28	b = 0.04 β = 2.21	b = −0.05	b = 0.07	b = −0.27	b = −1.03	b = 1.61
(short)	mid	2.22 ± 0.29	2.17 ± 0.26		β = −0.01	β = 0.02	β = −0.07	β = −0.28	β = 0.43
	end	2.21 ± 0.31	2.20 ± 0.25		*p* = 0.760	*p* = 0.960	*p* = 0.837	*p* = 0.500	*p* = 0.289
Trunk	begin	14.09 ± 1.65	14.34 ± 1.58	b = −0.18 β = 13.98	b = 0.24	b = −0.73	b = 0.30	b = 1.41	b = −0.40
(very short)	mid	13.93 ± 1.48	14.45 ± 2.01		β = 0.45	β = −1.39	β = 0.58	β = 2.68	β = −0.77
	end	13.91 ± 1.71	14.51 ± 2.05		*p* = 0.239	*p* = 0.341	*p* = 0.694	*p* = 0.116	*p* = 0.651
Lumbar spine	begin	12.24 ± 1.14	12.58 ± 1.31	b = −0.27 β = 12.17	b = 0.37	b = 0.21	b = 1.55	b = 1.09	b = −1.35
(very short)	mid	12.01 ± 1.40	12.67 ± 1.44		β = 0.52	β = 0.30	β = 2.17	β = 1.53	β = −1.90
	end	12.28 ± 1.53	12.82 ± 1.38		*p* = 0.066	*p* = 0.798	*p* = 0.066	*p* = 0.268	*p* = 0.171
Foot	begin	13.61 ± 1.10	14.44 ± 1.48	b = −0.50 β = 13.72	b = 0.68	b = 0.84	b = −0.39	b = 1.68	b = −0.13
(very short)	mid	13.76 ± 1.30	14.76 ± 1.37		β = 0.99	β = 1.22	β = −0.57	β = 2.45	β = −0.19
	end	13.78 ± 1.06	14.92 ± 1.48		*p* < 0.001	*p* = 0.304	*p* = 0.631	*p* = 0.078	*p* = 0.893

**Table 3:** LDE measures for PwMS with a varying level of disability. β: model parameter estimate after z-transformation of the LDE values.

	Model Estimates					
	Intercept	EDSS	Time	Time^2^	EDSS × Time	EDSS × Time^2^
Trunk	b = −0.42	b = 0.21	b = −0.38	b = −0.84	b = 0.41	b = 0.59
(short)	β = 0.91	β = 0.03	β = −0.06	β = −0.14	β = 0.07	β = 0.09
		*p* = 0.012	*p* = 0.772	*p* = 0.518	*p* = 0.471	*p* = 0.306
Lumbar spine	b = −0.46	b = 0.23	b = 0.25	b = −0.87	b = −0.14	b = 0.46
(short)	β = 1.00	β = 0.04	β = 0.04	β = −0.15	β = −0.02	β = 0.08
		*p* = 0.014	*p* = 0.825	*p* = 0.440	*p* = 0.776	*p* = 0.346
Foot	b = −0.22	b = 0.11	b = 0.54	b = 2.25	b = −0.70	b = −0.53
(short)	β = 2.14	β = 0.03	β = 0.15	β = 0.59	β = −0.18	β = −0.14
		*p* = 0.210	*p* = 0.645	*p* = 0.065	*p* = 0.188	*p* = 0.318
Trunk	b = −0.30	b = 0.15	b = −0.97	b = −1.00	b = 0.76	b = 0.46
(very short)	β = 13.83	β = 0.30	β = −1.92	β = −1.99	β = 1.51	β = 0.91
		*p* = 0.110	*p* = 0.184	*p* = 0.169	*p* = 0.018	*p* = 0.150
Lumbar spine	b = −0.26	b = 0.13	b = −1.22	b = −1.72	b = 1.17	b = 0.93
(very short)	β = 12.33	β = 0.18	β = −1.69	β = −2.38	β = 1.62	β = 1.29
		*p* = 0.178	*p* = 0.160	*p* = 0.049	*p* = 0.002	*p* = 0.014
Foot	b = −0.52	b = 0.26	b = 0.97	b = −1.67	b = 0.60	b = 0.61
(very short)	β = 13.95	β = 0.38	β = 1.41	β = −2.43	β = 0.87	β = 0.89
		*p* = 0.004	*p* = 0.271	*p* = 0.059	*p* = 0.120	*p* = 0.114

## Data Availability

The data that support the findings of this study are available on request from the corresponding author, R.M.
